# Indole Hydrazide Compound IHZ-1 Induces Apoptosis and Autophagy *via* Activation of ROS/JNK Pathway in Hepatocellular Carcinoma

**DOI:** 10.3389/fonc.2022.811747

**Published:** 2022-02-07

**Authors:** Manting Sun, Dan Liu, Yang Yuan, Juhua Dan, Shuting Jia, Ying Luo, Jing Liu

**Affiliations:** ^1^ Laboratory of Molecular Genetics of Aging and Tumor, Medical School, Kunming University of Science and Technology, Kunming, China; ^2^ Guizhou Provincial Key Laboratory of Pathogenesis & Drug Development on Common Chronic Diseases, School of Basic Medicine, Guizhou Medical University, Guiyang, China

**Keywords:** IHZ-1, JNK/ROS, autophagy, apoptosis, hepatic cellular carcinoma (HCC)

## Abstract

Hepatocellular carcinoma is one of the most common primary malignant tumors of the digestive system. Compound 5-chloro-*N*′-(2,4-dimethoxybenzylidene)-1*H*-indole-2-carbohydrazide (IHZ-1/ZJQ-24) is a novel indole hydrazide derivative. In a recent study, we demonstrated that IHZ-1 inhibits tumor growth and induces cell apoptosis through inhibiting the kinase activity of mTORC1 without activation of AKT, which is associated with JNK/IRS-1 activation. However, the impact and mechanisms of JNK activation by IHZ-1 in hepatocellular carcinoma remains entirely unknown. Here, we find that IHZ-1 increases the generation of intracellular ROS and enhances autophagy. The phosphorylation of JNK induced by IHZ-1 was reversed by the decreased ROS level. Moreover, inhibition of ROS/JNK or autophagy equally attenuated apoptotic effect induced by IHZ-1. Our findings suggest that the activation of JNK by IHZ-1 treatment is dependent on the generation of ROS that mediates apoptosis and autophagy in hepatocellular carcinoma.

## Introduction

Primary carcinoma of the liver [hepatocellular carcinoma (HCC)] is one of the familiar digestive system neoplasms (1). Current treatments for HCC include surgery, radiation therapy, and drug therapy, but the overall outlook for most patients has not improved significantly, with 5-year survival rates below 20% (2). Therefore, the development of novel therapies for the management of HCC is especially urgent.

**Figure 1:**
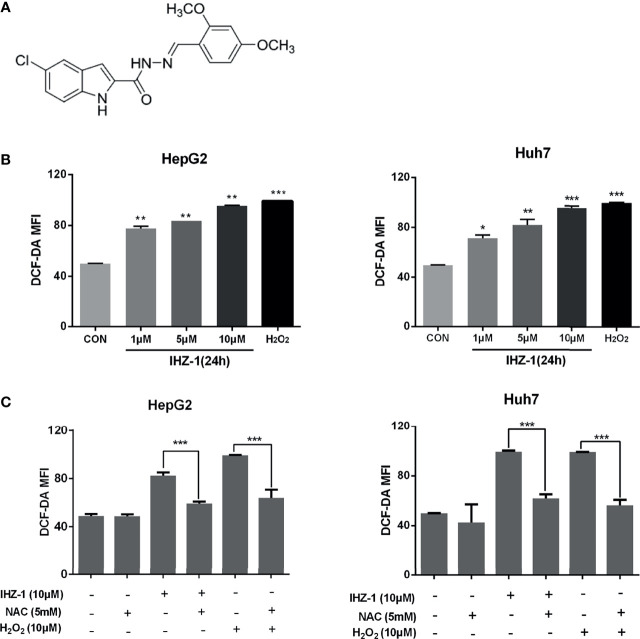
IHZ-1 induced ROS generation in HCC cells. **(A)** Structural formula of compound IHZ-1. **(B)** Intracellular ROS level were measured by fluorescence microscope with DCFH-DA staining after IHZ-1 treatment **(C)** or pretreated with NAC and analyzed by flow cytometer. *p < 0.05, **p < 0.01, and ***p < 0.001.

Our previous study reported that IHZ-1 has also been named ZJQ-24 (5-chloro-*N*′-(2,4-dimethoxybenzylidene)-1H-indole-2-carbohydrazide) (**Figure 1A**) induced cell apoptosis by inhibiting the mTORC1 activity without activation of AKT *via* phosphorylation of c-Jun N-terminal kinase (JNK) (3). Although the inhibition of AKT/mTOR by IHZ-1 has positive correlation with antitumor effect, the mechanisms of JNK activation remain unclear. Also, whether the activation of JNK by IHZ-1 can result in apoptosis or autophagy has not been investigated yet.

JNK is a type of serine/threonine kinase which belongs to one of the mitogen-activated protein kinases (MAPK), and its activation regulates many cellular progresses, such as apoptosis and autophagy which are two main types of programmed cell death (PCD) (4). In the early transient phase of JNK activation, JNK serves to promote autophagy and cell survival because of upstream protein kinase of Bcl-2 family proteins (5). Whereas in the later and more sustained phase of JNK activation, JNK serves to induce apoptosis (6). For several decades, apoptosis has been considered the pivotal mechanism of cell death in tumor cells *via* mitochondria-dependent pathway (7), and enhancing cell apoptosis is also a main strategy of cancer therapy. However, many studies have reported that the mechanisms of anticancer treatment are not only confined to apoptosis but also involved autophagy. Autophagy has been identified as a “self-eating” process of organelles and cytosolic macromolecules (8). It plays an important role in cell death, normal physiology, and cellular homeostasis. However, autophagy is defined as type II programmed cell death and has a dual role including prodeath or prosurvival which depends on the cell type and the level of stress (9). Although, the crosstalk between autophagy and apoptosis is very complicated and remains controversial. They may be triggered by common upstream stressor reactive oxygen species (ROS) which is known as a mediator for the activation of MAPK (10). In cancer research, increasing studies suggest that different levels of ROS results in autophagy and apoptosis (11). Hence, targeted activation on the ROS/JNK pathway might be beneficial in antitumor treatment. Here, we found the activation of JNK by IHZ-1 was dependent on ROS generation and induced cell apoptosis. Furthermore, we investigated the IHZ-1-induced cell apoptosis and autophagy regulated by the ROS/JNK signaling pathway.

## Materials and Methods

### Chemicals

IHZ-1 compound was identified and provided by the Key Laboratory of Medicinal Chemistry for Natural Resource (Yunnan University). The compound was prepared as 100 mM stock solutions in DMSO and aliquots stored at −20°C, protected from light. Reagents, unless specified otherwise, were purchased from Sigma-Aldrich Ltd. (Shanghai, China).

### Cell Lines and Culture Conditions

The cell lines (HepG2, HUH-7) were obtained from the American Type Culture Collection (ATCC, Manassas, VA, USA). Cells were cultured in DMEM medium (Gibco, Invitrogen, New York, NY, USA) supplemented with 10% FBS (Gibco) at 37°C in a humidified atmosphere of 5% CO_2_.

### ROS Detection

ROS level was measured by 2,7-dichlorofluo-rescein diacetate (DCFH-DA). Cells on a 96-well plate were allowed to attach overnight, then treated with IHZ-1 (1, 5, and 10 μM) for 24 h. The medium was removed and washed 3 times with PBS, then incubated with DCFH-DA at 37°C for 30 min, measuring the ROS levels through fluorescence microplate reader with 488 nm excitation wavelength and 525 nm emission wavelength.

### Polymerase Chain Reaction (Real-Time PCR)

Real-time PCR analysis was performed as described previously (12). Total RNA was extracted using TRIzol reagent (Invitrogen), then 500 ng total RNA was reversely transcribed into cDNA by High-Capacity cDNA Reverse Transcription Kit (Applied Biosystems, Waltham, MA, USA). The mRNA expression was detected using SYBR Green assay in 7300 Real-Time PCR System (Applied Biosystems). The following primers were used: *Atg5* forward 5′-CACTTTGTCAGTTACCAACGTCA-3′ and *Atg5* reverse 5′-AAAGATGTGCTTCGAGATGTGT-3′; *ULK1* forward 5′-CGACCTCCAAATCGTGCTTCT-3′ and *ULK1* reverse 5′-GGCAAGTTCGAGTTCTCCCG-3′; and *Beclin1* forward 5′-GAATCTGCGAGAGACACCATC-3′ and *Beclin1* reverse 5′-CCATGCAGGTGAGCTTCGT-3′. The program of real-time system is 95°C for 10 min, then 40 cycles of 95°C for 15 s and 60°C for 1 min. Data analysis was performed using the following equations: △C=Ct(sample)−Ct(endogenous control); △△ Ct=△Ct(sample)−Ct(untreated); and fold change = 2^−△△Ct^.

### GFP-LC3-II Transfection

HepG2 and Huh7 cells were seeded onto cover slips in 6-well plates at a density of 1 × 10^5^ cells/well and left overnight, then transfected with 3 μg GFP-LC3-II plasmid using Lipo2000 (Invitrogen Corporation) according to manufacturer’s instructions. After 24 h, cells were treated with IHZ-1 (1, 5, and 10 μM) and vehicle. After 24 h, cells were fixed with 4% paraformaldehyde in PBS at 4°C for 30 min, and GFP-LC3-II fluorescence was examined under a fluorescence microscope. For each slide, 100 cells were analyzed and mean numbers of punctate GFP-LC3-II spots/cell were calculated.

### Western Blot Analysis

For each sample, all the cells were lysed for 30 min in lyses buffer on 4°C; after ultrasound, the cell debris was centrifuged at 12,000 rpm for 20 min at 4°C, protein concentration was detected by BCA (Beyotime, Haimen, China), using 12% sodium dodecyl sulfate-polyacrylamide gel electrophoresis (SDS-PAGE) to separate the proteins, the separated proteins were blotted onto PVDF membrane, blocking 2 h at room temperature with 5% nonfat dry milk in TBST, incubating the antibody for 16 h at 4°C with 1:1,000 concentration, washing PVDF membrane 3 times for 10 min in TBST, incubating secondary antibody at 25°C for 2 h with the 1:10,000 concentration, washing membranes 3 times for 10 min in TBST. Finally, the membranes were visualized with the enhanced chemiluminescence (ECL) detection system (GE Healthcare, Piscataway, NJ, USA). All of the primary antibodies (p-JNK, JNK, P62, LC3-II, GAPDH) were purchased from Cell Signaling Technology, Inc. (Danvers, MA, USA).

### Annexin-V/PI Assay

Apoptotic cells were identified using Annexin-V/PI staining analyses. Briefly, cells were respectively seeded into 6-well plates and treated with IHZ-1 in different concentrations (1, 5, and 10 µM) or IHZ-1combine with chloroquine (10 μM) for 24 h. Cells were then harvested and washed using PBS. The surface levels of phosphatidylserine were quantitatively detected using Annexin V-FITC and PI apoptosis kit according to the manufacturer’s instructions (BD Biosciences, San Jose, CA, USA). The stained cell number were analyzed by flow cytometry (Accuri C6, BD Biosciences, Franklin Lakes, NJ, USA) within 1 h.

### Quantification of Acidic Vesicular Organelles With Acridine Orange

HepG2 and Huh7 cells were seeded into 6-well plates and allowed to attach overnight before treatment with IHZ-1(1, 5, and 10 μM) and vehicle. Cells were stained with acridine orange (AO) (1 mg/ml) for 15 min. Media were removed and cells washed with PBS and analyzed by flow cytometry, green (510–530 nm, FL1-H channel) and red (>650 nm, FL3-H channel). The bar for FL3-H in the control sample was set so that the acidic vesicular organelle (AVO)-positive cells represented approximately 5% of the population. Compound-treated samples were measured under the same conditions.

### Statistical Analysis

The results were expressed as mean ± standard deviation (SD). Statistical differences were evaluated using the two-tailed Student’s *t*-test and analysis of variance (ANOVA) followed by *q*-test, considered significant at ^*^p < 0.05, ^**^p < 0.01, or ^***^p < 0.001.

## Results

### IHZ-1 Activation of JNK *via* ROS Generation To Induce Cell Apoptosis

For research if whether IHZ-1 induces the upregulation of ROS, we used 2,7-dichlorodihydro-fluorescein diacetate (DCFH-DA) staining followed by flow cytometry; H_2_O_2_ was included as a positive control. As shown in **Figure 1B**, IHZ-1 induced ROS generation in a dose-dependent manner, as reflected by the increase of fluorescence intensity. To further verify that IHZ-1 induces the generation of ROS, we used *N*-acetylcysteine (NAC) to clear ROS and detect changes in ROS levels. The data showed that the high ROS levels dropped after NAC treatment (**Figure 1C**).

**Figure 2:**
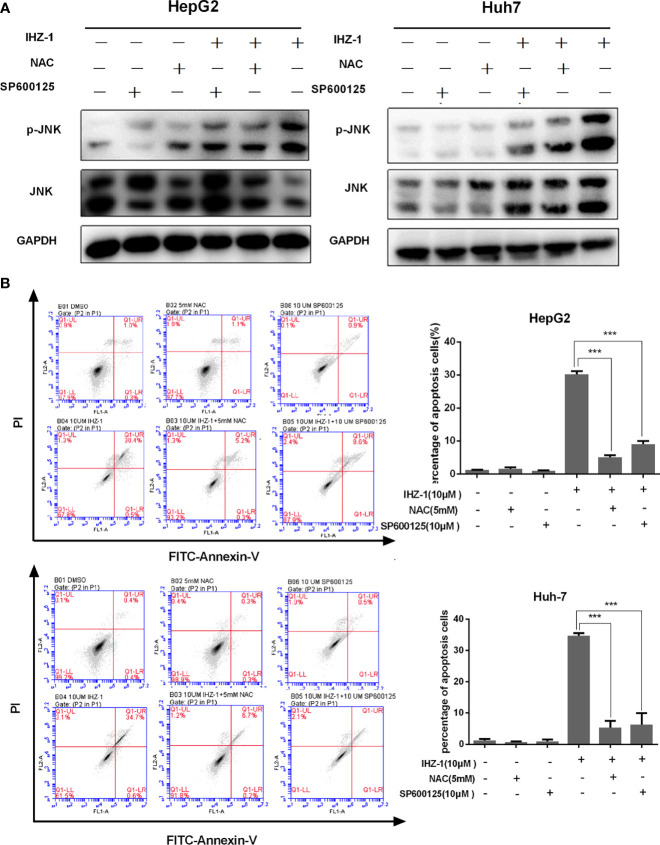
IHZ-1 caused apoptosis through activating ROS/JNK cascade in HCC cells. **(A)** Cells were incubated with IHZ-1 and pretreated with NAC or SP600125. Immunoblots of p-JNK and JNK protein expression was quantified. **(B)** The apoptosis cells were evaluated by flow cytometry using Annexin-V/PI double staining. ***p < 0.001.

It is reported that JNK can be regulated by ROS (13). The production of ROS easily activates JNK, which usually plays a very important role for cell survival (14, 15). So we treated cells with NAC and JNK inhibitor (SP600125) and tested the expression of JNK in HCC cells. Our result showed that IHZ-1 increased the expression level of p-JNK and the upregulation of p-JNK was inhibited by NAC and SP600125 (**Figure 2A**). To demonstrate the effect of ROS-induced JNK activation on cell survival, we used flow cytometry to detect apoptosis by Annexin-V/PI double staining. When ROS and JNK were inhibited, the number of apoptotic cells was reduced (**Figure 2B**). Collectively, we demonstrated that IHZ-1 caused JNK upregulation in ROS-dependent HCC cells, which is required for IHZ-1-induced apoptosis.

### IHZ-1 Triggers Autophagy

**Figure 3:**
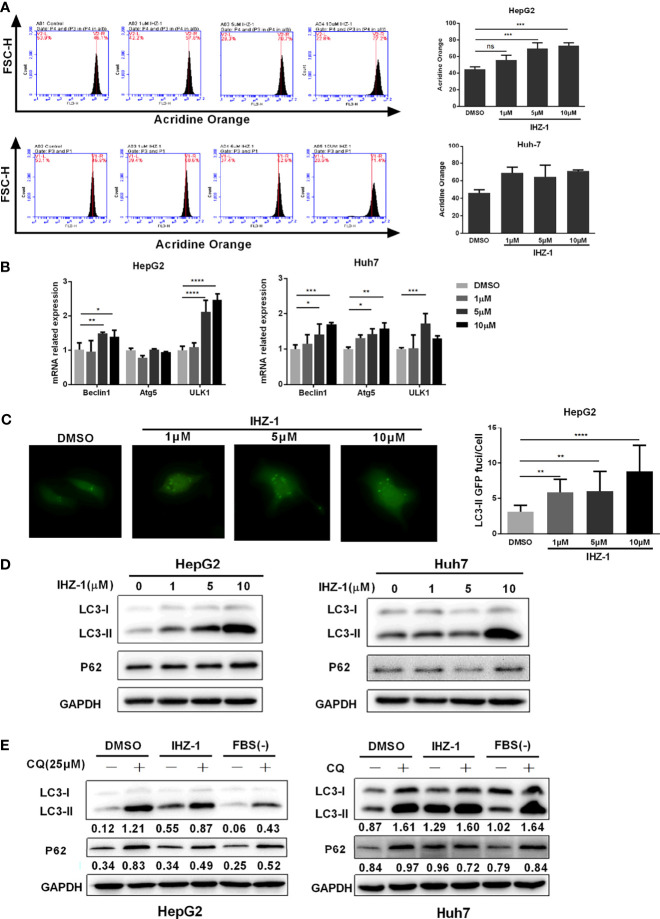
IHZ-1 triggers autophagy. **(A)** The number of acidic organelles were evaluated by acridine orange staining and analyzed by flow cytometry. **(B)** The autophagy-related mRNAs were determined by real-time PCR. **(C)** Representative images of HCC cells steadily articulating GFP-LC3 after IHZ-1 treatment. **(D)** The autophagy-related proteins were determined by western blotting. **(E)** The autophagic flux was determined by immunoblot analysis of LC3-II and P62 levels in HCC cells incubated with IHZ-1 in the presence or absence of CQ. As indicated below each lane, the LC3-II/GAPDH and P62/GAPDH ratios were determined using the ImageJ software. *p < 0.05, **p < 0.01, ***p < 0.001, and ****p < 0.0001. no significance (ns).

Recent studies have shown that ROS-mediated autophagy has two opposing functions in a variety of HCC treatments: promoting cell survival or inducing cell death (16). Therefore, we first used AO staining to evaluate the number of acidic organelles in HepG2 and Huh7 cells. As shown in **Figure 3A**, the number of acidic organelles increased especially in HepG2 cells. In addition, the real-time PCR showed that the mRNA expression levels of *Beclin1*, *Atg5*, and *ULK1* increased after IHZ-1 treatment (**Figure 3B**). To further determine the activation of autophagy, the GFP-LC3II lentivirus was transfected in HepG2 cells to detect the fluorescent puncta formation of autophafosomes. After treatment with IHZ-1 for 24 h, green puncta formation presented a significant increase in a dose-dependent manner (**Figure 3C**). Next, Western blotting assay was used to test the LC3-II and p62 which are the main marker proteins of autophagy. It showed that IHZ-1 treatment upregulated the LC3-II in HCC cells. However, the amount of p62 that was delivered to the lysosomes for degradation did not decrease after IHZ-1 treatment (**Figure 3D**). This result seems to suggest that IHZ-1 impairs the autophagic degradation process. In order to obtain a better evaluation of autophagic flux, we carried out Western blotting to detect the p62 expression using the cells treated with autophagy inhibitor chloroquine (CQ, 25 μM) as control (17). We also used the starvation medium as a positive control to induce the autophagic flux. As shown in **Figure 3E**, IHZ-1 decreased the level of p62 in the presence of CQ. These results suggested that IHZ-1 caused autophagy through activating ROS/JNK in HCC cells.

### Suppression of the Autophagy Flux Enhanced by IHZ-1 Promotes Apoptosis

**Figure 4:**
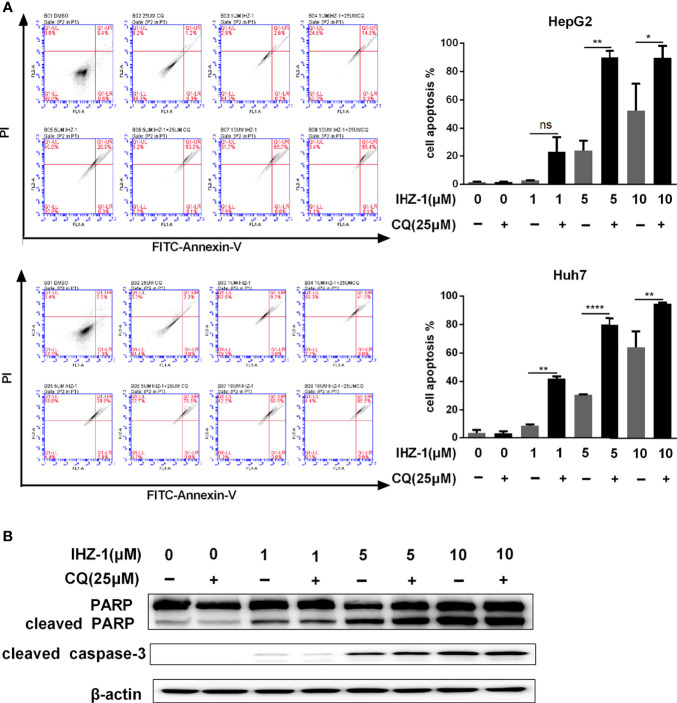
Suppression the autophagy flux enhanced by IHZ-1 promote apoptosis. **(A)** The autophagic flux induced by IHZ-1 was inhibited by CQ. The apoptotic cells were then evaluated by flow cytometry with Annexin-V/PI staining. **(B)** The apoptosis-related protein levels were determined by Western blotting. *p < 0.05, **p < 0.01, and ****p < 0.0001. no significance (ns).

Autophagy has opposite roles for therapeutic purposes in tumor, including the effect of promoting or preventing apoptotic cell death. We further used CQ to inhibit IHZ-1-induced autophagy in HCC cells. Flow cytometric analysis results showed that the administration with CQ significantly enhance the apoptosis effect induced by IHZ-1 (**Figure 4A**). In addition, Western blotting results revealed that treatment with CQ also strengthened apoptosis-associated protein expression induced by IHZ-1, such as cleaved PARP and cleaved caspase3 (**Figure 4B**). The above results indicated that IHZ-1 triggered autophagy of HCC in a dose-dependent manner. The autophagy triggered by IHZ-1 may be antiapoptosis.

## Discussion

**Figure 5:**
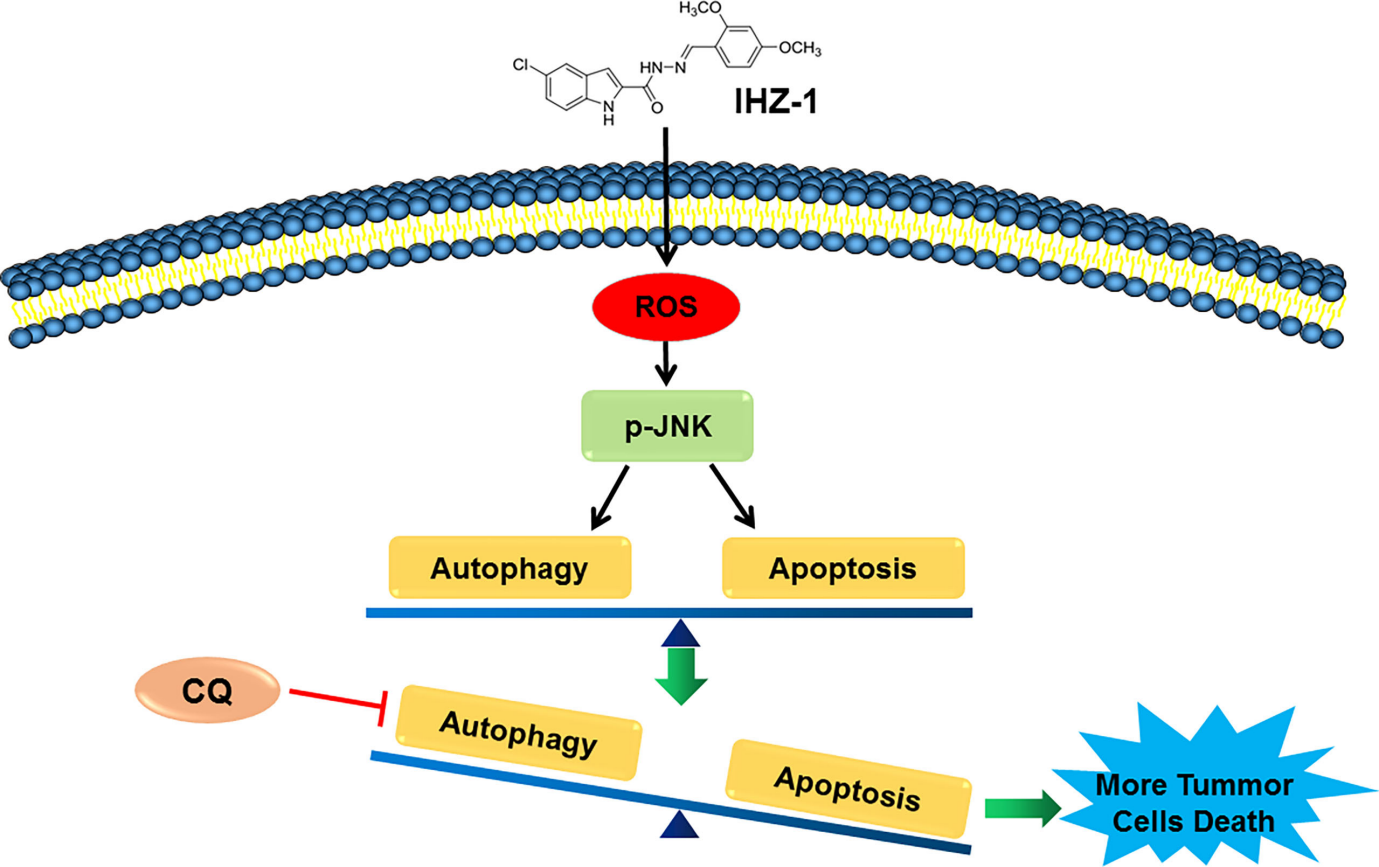
The mechanism of IHZ-1 induced apoptosis and autophagy in HCC cells. IHZ-1 could accumulate ROS and induce autophagy in HCC cells, while inhibiting autophagy could enhance the apoptosis induced by IHZ-1.

Our previous study has reported that IHZ-1 (ZJQ-24) effectively suppressed HCC proliferation *via* G_2_/M phase arrest and caspase-dependent apoptosis. IHZ-1 induced cell apoptosis by inhibiting the mTORC1 activity without activation of AKT *via* phosphorylation of JNK (3). However, the mechanisms of JNK activation remain unclear. Here, we have detected that JNK activation by IHZ-1 is ROS dependent which results in cell apoptosis. Further experiments indicated that the ROS generation by IHZ-1 also induces autophagy. In addition, CQ could strengthen the power of IHZ-1-induced apoptosis, which suggested that the autophagy induced by IHZ-1 may support cell survival (**Figure 5**).

Accumulated evidence has indicated that JNK activation could transduce oxidative stress and induce cell apoptosis (18, 19). This guided us to believe that the JNK activation by IHZ-1 was ROS dependent. A high level of ROS after IHZ-1 treatment in HCC cells was found during the research of apoptosis. In addition, we found that accumulation of ROS could trigger the activation of JNK, whereas pretreatment with NAC could attenuate the phosphorylation of JNK. Our data specified that IHZ-1 may active the JNK by induction of the high level of ROS. In this study, we also found that the apoptosis induced by IHZ-1 was attenuated by pretreatment with NAC and SP600125, a JNK inhibitor. Taken together, these results showed that IHZ-1 induces apoptosis by triggering the ROS/JNK-dependent pathway.

Besides apoptosis, ROS/JNK pathway activation could also induce autophagy, which also has a vital role in regulating cell death. During the process of autophagy, the cytoplasmatic vesicles and intracellular organelles were transported into autophagodomes, then decomposed and digested in the lysosome (20). Accumulated studies indicates that autophagy critically regulates cellular homeostasis and has two-edged sword effects, providing protection or causing cell damage (21). In our study, we first identified that IHZ-1 could induce autophagy with the results that the number of acidic organelles was increased and the expression levels of autophagy genes and LC3-II were enhanced. To further determine the autophagic flux, we tested the LC3-II and p62 levels by immunoblotting in the presence of CQ. Our data demonstrated that IHZ-1 attenuated the expression of p62 after CQ treatment, suggesting the activation of autophagic flux. Our results also indicated that the autophagy inhibitor CQ could enhance the power of IHZ-1-induced apoptosis, which suggested that autophagy triggered by IHZ-1 may support survival during the death of HCC cells.

Some evidence has demonstrated that ROS was a signaling molecule; it could induce apoptosis and/or activate autophagy, depending on the cellular components (22). Meanwhile, apoptosis and autophagy share many signaling pathways, such as the MAPK pathway including JNK, p38 MAPK, and ERK (23). However, the molecular mechanisms of ROS/JNK-mediated apoptosis and autophagy with IHZ-1 treatment are still not clear. It is worthy to note in our study that IHZ-1 could increase generation of ROS and activate ROS-dependent JNK signaling pathway, while the cell fate of IHZ-1 on cell apoptosis and autophagy was opposite. It suggested that autophagy could be a self-protective reaction of tumor cells with IHZ-1 treatment. Hence, we speculated that triggering apoptosis and autophagy by IHZ-1 was associated with the level of ROS.

In conclusion, our results showed that IHZ-1 could significantly induce apoptosis and autophagy through activation of ROS-dependent JNK signaling pathways. Furthermore, inhibition autophagy enhanced IHZ-1-induced apoptosis, suggesting that IHZ-1-induced autophagy played a protective role in HCC cells. These findings not only indicated that IHZ-1 induced apoptosis through ROS/JNK pathways but also offer an alternate approach of combining IHZ-1 with autophagy inhibitor together for human HCC treatment as well.

## Data Availability Statement

The original contributions presented in the study are included in the article/supplementary material. Further inquiries can be directed to the corresponding authors.

## Author Contributions

All the authors confirmed that they have reviewed the manuscript entitled “Indole Hydrazide Compound IHZ-1 Induces Apoptosis and Autophagy *via* Activation of ROS/JNK Pathway in Hepatocellular Carcinoma” and approved its submission to the journal of “Frontiers in Oncology”. The specific work of each author in this study was as follows: SJ, YL, and JL: perception and design and final approval of the version to be published. MS, DL, YY, and JD: participation in the whole work, drafting of the article, and data analysis.

## Funding

This study was supported by grants from the National Natural Science Foundation of China (No. 82060660) and Yunnan Fundamental Research Project (grant No. 202101AT070086, 2019FB109).

## Conflict of Interest

The authors declare that the research was conducted in the absence of any commercial or financial relationships that could be construed as a potential conflict of interest.

## Publisher’s Note

All claims expressed in this article are solely those of the authors and do not necessarily represent those of their affiliated organizations, or those of the publisher, the editors and the reviewers. Any product that may be evaluated in this article, or claim that may be made by its manufacturer, is not guaranteed or endorsed by the publisher.
